# A framework of evolutionary optimized convolutional neural network for classification of shang and chow dynasties bronze decorative patterns

**DOI:** 10.1371/journal.pone.0293517

**Published:** 2024-05-14

**Authors:** XiuZhi Qi, XueMei He, Shan Wei Chen, Tao Hai

**Affiliations:** 1 College of Art and Design, Shaanxi University of Science & Technology, Xi’an, China; 2 Academy of Fine Arts, Baoji University of Arts and Sciences, Baoji, China; 3 Faculty of Art, Computing and Creative Industry, Universiti Pendidikan Sultan Idris, Tanjong Malim, Perak, Malaysia; 4 Department of Education, Baoji University of Arts and Sciences, Baoji, China; 5 School of Computer and Information, Qiannan Normal University for Nationalities, Guizhou, China; 6 Institute for Big Data Analytics and Artificial Intelligence (IBDAAI), Universiti Teknologi MARA, Shah Selangor, Malaysia; National University of Sciences and Technology NUST, PAKISTAN

## Abstract

As a UNESCO World Cultural Heritage, the aesthetic value of bronze artifacts from the Shang and Chow Dynasties has had a profound influence on Chinese traditional culture and art. To facilitate the digital preservation and protection of these Shang and Chow bronze artifacts (SCB), it becomes imperative to categorize their decorative patterns. Therefore, a SCB pattern classification method of differential evolution called Shang and Chow Bronze Convolutional Neural Network (SCB-CNN) is proposed. Firstly, the original bronze decorative patterns of Shang and Chow dynasties are collected, and the samples are expanded through image augmentation technology to form a training dataset. Secondly, based on the classical convolutional neural network structure, the recognition and classification of bronze patterns are implemented by adjusting the network parameters. Then, the initial parameters of the convolutional neural network are optimized by differential evolution algorithm, and the optimized SCB-CNN is simulated. Finally, comparative experiments were conducted between the optimized SCB-CNN, the unoptimized model, VGG-Net, and GoogleNet. The experimental results indicate that the optimized SCB-CNN significantly reduces training time while maintaining fast prediction speed, convergence speed, and high accuracy. This study provides new insights for the inheritance and innovation research of SCB patterns.

## 1 Introduction

Over a span of more than 1,000 years, Chinese bronze art underwent significant evolution during the Xia Dynasty, Shang Dynasty, Chow Dynasty, Spring and Autumn period, and Warring States period, culminating in the development of a distinctive bronze culture [1]. Among these, the Shang and Chow Dynasty bronzes (SCB) stand as a remarkable testament to the technological and artistic advancements of their respective eras, representing a vital facet of ancient Chinese bronze craftsmanship, simultaneously, they are considered an essential component of the world’s cultural heritage [2].

As a significant component of China’s culture, the exquisite patterns on SCB carry a wealth of historical information and cultural significance. They document the religious practices, rituals, lifestyles, and technological advancements of ancient societies. The classification and preservation of these patterns contribute to the better inheritance of China’s cultural heritage. SCB not only holds artistic value but also serves as a valuable resource for historical research. Through the classification and study of these patterns on bronze artifacts, a deeper understanding of information related to ancient society’s religious practices, rituals, and lifestyles can be gained. This holds immense value for historians, archaeologists, and cultural researchers. The patterns on SCB possess a strong cultural emblem, representing the unique characteristics of ancient Chinese culture. They hold significant importance for national and ethnic identity, and preserving these patterns is a way of upholding the nation’s cultural identity [3].

Researching the pattern classification of SCB not only contributes to the preservation and inheritance of cultural heritage but also provides a wealth of resources and opportunities for digital innovation [4]. The digital preservation and innovation of SCB encompass various facets, including collection and storage, restoration and reproduction, display and dissemination, as well as the development and enhancement of pattern images [5]. These intricate bronze patterns can be categorized into three main types: animal motifs, geometric designs, and depictions of human activities. Animal patterns encompass beast faces, dragons, phoenixes, and curvilinear motifs. Geometric patterns include cloud and thunder motifs, strings, linked circles, and breast patterns. Human activity patterns feature dances, hunts, and war scenes [6]. These patterns comprise essential elements–points, lines, and surfaces–which can be scaled, rotated, and locally deformed to create diverse motifs [7].

Currently, the field of SCB pattern classification faces several significant research shortcomings and challenges, which hinder the progress of digital preservation and innovation of this precious cultural heritage. Firstly, the diverse array of patterns on SCB, including animals, geometric designs, and depictions of human activities, each with multiple subcategories, presents a highly challenging task for pattern classification and recognition. Traditional image processing techniques and rule-based methods often struggle to handle this complexity effectively [8]. Secondly, acquiring a substantial amount of SCB image data and accurately annotating it is a monumental challenge. Original SCB artifacts are scattered across museums and private collections worldwide, necessitating international cooperation for access. Furthermore, precise annotation of these images demands expertise and considerable time, making data annotation a time-consuming and expensive task [9]. Thirdly, due to the limited availability of SCB image data, overfitting is a common issue when training deep learning models. Hence, it’s essential to find suitable methods to effectively augment the limited dataset and improve the model’s performance [10]. Fourthly, traditional deep learning models are overly complex, resulting in extended training times and high computational resource demands. Additionally, these complex models lack interpretability, making it difficult to understand the decision-making process behind pattern classification.

The SCB Convolutional Neural Networks (SCB-CNN) method proposed in this paper is based on deep learning technology, aiming to address the challenges of SCB pattern classification. The design rationale and foundation for this approach are as follows:

Firstly, patterns on SCB are typically highly intricate, encompassing a plethora of details and diversity. Traditional classification methods often rely on human experts, which can be limiting when dealing with complex and varied patterns. Deep learning techniques, exemplified by Convolutional Neural Networks (CNNs), are renowned for their outstanding performance in processing complex image data, making them well-suited to handle the complexity and diversity of SCB patterns [11]. Secondly, deep learning is a data-driven approach capable of learning valuable features and patterns from large-scale datasets [12]. Given that SCB pattern classification needs to consider various types of patterns, deep learning can learn the commonalities and differences among these patterns from a vast amount of image data. This data-driven approach allows for better adaptation to different types of SCB patterns. Thirdly, CNNs can automatically learn and extract features from images without the need for manually designed feature extractors [13]. This is particularly advantageous for SCB pattern classification since different types of patterns require different feature representations. CNNs can automatically adapt to these features, thereby enhancing classification accuracy. Fourthly, by utilizing pre-trained CNN models, it’s possible to leverage model parameters trained on large-scale image datasets, reducing the time and resource costs of model training [14]. This transfer learning approach makes SCB pattern classification models easier to train and optimize. Fifthly, to enhance the model’s robustness and performance, the study employs data augmentation techniques, including image scaling, rotation, cropping, and more. These techniques help expand the training dataset, mitigate the risk of overfitting, and improve the model’s generalization capability.

The SCB-CNN method proposed in this paper boasts several advantages, all of which have been thoroughly demonstrated in the experimental and analytical sections of the article. Firstly, the SCB-CNN model exhibits a high level of accuracy in pattern classification tasks, with an average P+ reaching 98.8%. This signifies the model’s ability to accurately classify SCB patterns into their correct categories, a critical aspect of cultural heritage preservation. Secondly, the optimized SCB-CNN model significantly reduces training time, decreasing from the original 1354.48 seconds to 262.67 seconds. This reduction implies that the model can converge faster within the same number of training epochs, enhancing research efficiency. Thirdly, the incorporation of data augmentation techniques, such as image scaling, rotation, and cropping, contributes to improved model robustness, mitigating the risk of overfitting. This allows the model to maintain good performance when handling patterns of different sizes, angles, and transformations. Lastly, comparative experiments with other deep learning models like VGG-Net and GoogleNet reveal that SCB-CNN competes favorably in terms of training time, model convergence speed, and accuracy. It even surpasses some traditional methods and deep learning models in these aspects. These advantages position SCB-CNN as a promising approach with the potential to play a significant role in cultural heritage preservation and digital innovation.

The remainder of this paper is structured as follows: Section 2 reviews and analyzes current research progress in image recognition and classification, particularly in the context of pattern classification and cultural heritage preservation. Section 3 outlines the dataset and details the application of evolutionary optimization in customizing convolutional neural networks for classification. Section 4 offers a comprehensive description of the experimental implementation, and summarizes and analyzes the experimental results, including comparisons with other models. Finally, Section 5 concludes by summarizing key findings, and contributions, and suggesting improvements and potential future research directions.

## 2 Related work

Image classification is a fundamental and significant topic in the field of computer vision. Its fundamental purpose is to divide images into multiple predetermined categories based on their unique characteristics [15]. In recent years, there has been significant progress in the field of image classification research with deep learning methods [16]. The deep learning method is a perceptron learning network with several hidden layers [17]. Through automatic learning, it feeds back and optimizes the appropriate image bottom features and automatically combines and abstracts the bottom features in the deep stage to form a deep representation.

In this realm, Sanjiban Sekhar Roy et al. introduced a dilated CNN approach for urban sound classification. According to the results, an incremental dilation rate was employed in this study, achieving an accuracy of 84.16% with the proposed dilated convolutional method [18]. Zheng et al. proposed a two-level data augmentation method in deep learning based on spectrum interference for automatic modulation classification. This approach initially employs short-time Fourier transform to shift the original signal into the frequency domain, and subsequently feeds both the original and augmented signals into the network. Ultimately, by utilizing data augmentation, they enhance the generalization performance of the deep neural network. Additionally, the study provides further evidence that data augmentation during the testing phase can be interpreted as model ensemble [19]. Zheng et al. introduced manifold regularization (MR) into an autoencoder (AE) for the first time, aiming to enhance cross-layer manifold invariance. They have devised a manifold regularization-based deep convolutional autoencoder (MR-DCAE) model for unauthorized broadcast recognition. This model utilizes a specially designed autoencoder (AE), optimized through entropy-stochastic gradient descent, and employs reconstruction error to determine whether the received signal is authorized. Comparative experiments demonstrate that MR-DCAE achieves state-of-the-art performance [20]. Mohammad Momeny et al. presents a Noise-Robust Convolutional Neural Network (NR-CNN) designed to classify noisy images without prior noise removal preprocessing, addressing noise issues at various network levels. Experimental results confirm that NR-CNN significantly improves noisy image classification and network training speed [21]. Zheng et al. introduced a multi-scale radio transformer (Ms-RaT) with a dual-channel representation for fine-grained modulation classification (FMC). During the learning process, this model incorporates multi-scale analysis to establish tighter decision boundaries. Simulation experiments demonstrate that compared to existing state-of-the-art deep learning methods, Ms-RaT achieves superior modulation classification accuracy with similar or lower computational complexity[22]. S. Yang et al. proposed a gradient-guided evolutionary approach to training deep neural networks (DNNs). This approach combines the advantages of both gradient-based methods and evolutionary algorithms. It suggests a novel genetic operator to optimize weights in the search space, where the search direction is determined by the gradient of the weights. Since network sparsity is considered in the proposed approach, it significantly reduces network complexity and mitigates overfitting. Experimental results on single-layer NNs, deep-layer NNs, recurrent NNs, and convolutional NNs (CNNs) demonstrate the effectiveness of the proposed approach [23]. Zheng et al. employed a pruning technique called Drop-path to reduce the size of a model based on the generalization error boundary. This method targets the reduction of parameters in 2D deep CNN models, ultimately accelerating network inference. Experimental results demonstrate that Drop-path achieves significant model compression and acceleration without any notable loss in accuracy[24]. Raj Biswas et al. proposed an architecture that employs dilated convolutional filters to obtain a larger receptive field, resulting in greater accuracy in segmenting retinal blood vessels, achieving near-human accuracy. The experimental results show that the proposed architecture produced an area under the ROC curve (AUC) of 0.9794 and an accuracy of 95.61%, requiring very few iterations to train the network [25]. Fatemeh Zare Mehrjardi et al. provides a comprehensive survey of image forgery detection with a focus on deep learning-based methods. This survey serves as a valuable resource for researchers seeking in-depth knowledge in the field of forgery detection [26]. Zheng et al. proposed a priori regularization method in deep learning (DL-PR) which regularizes deep learning models based on the signal-to-noise ratio (SNR) distribution of samples. This method guides the loss optimization during model training. The final experimental results demonstrate the superiority of DL-PR, with CNN achieving an accuracy of 62.6% and an inference time of 0.82 ms per signal, LSTM with 61.8% and 0.87 ms, and hybrid CNN–LSTM with 64.2% and 0.94 ms [27].

In recent years, CNN have also been employed in decoration pattern recognition and detection. Zhou et al. introduced an improved VGGNET classification model to enhance the classification accuracy of batik patterns. The classification accuracy of this model on the batik testing set can reach 92.12% and 95.79% [28]. Additionally, Li Zhi proposed a batik multicolor dyeing method based on CNN. This method can simulate the multi-color dyeing of images, significantly improving the halo dyeing effect compared to previous methods, making it closer to real batik patterns [29]. Liu Ying introduced a tire pattern image classification algorithm based on migration learning and feature fusion. This method incorporates migration learning into CNN model training, fine-tuning the pre-training model parameters using a tire image dataset. It obtains a new model suitable for tire pattern images and resolves the problem of CNN overfitting due to insufficient training data [30].

Aladine Chetouani et al. presented a method that classifies patterns of ceramic sherds by combining deep learning-based features extracted from some pre-trained CNN models. This approach was evaluated with a dataset composed of 888 digital patterns extracted from 3D scans of pottery sherds. The results show that the best result was obtained when features of the VGG19 and ResNet50 models were combined using Compact Bilinear Pooling (CBP) with a high classification rate of 95.23% [31].

Some studies have proposed improvement methods for pattern recognition in different domains. For example, CSV-Net is used to recognize patterns in traditional gardens in Suzhou [32]. Wang Fei and his team enhanced the ALEXNET model for recognizing cashmere and wool [33]. Jia Xiaojun and colleagues introduced improvements to the VGGNET model for blue calico pattern classification [34]. Jin Yamanaka and others presented a Single Image Super-Resolution (SISR) model based on Deep CNN, which offers lower computational costs compared to Deep CNN and delivers more advanced reconstruction performance [35].

While CNN have been widely applied, it is relatively rare to see their application in processing bronze patterns from the Shang and Chow dynasties. In this study, we aimed to collect a large number of SCB patterns and obtain numerous basic units from original samples with the assistance of a pattern extraction algorithm to establish CNN. Subsequently, we conducted automatic classification training on these basic elements. Based on the characteristics of the pattern’s basic unit, coupled with the differential evolution algorithm, we further improved and optimized the parameters of the CNN. Finally, we established a CNN classification model for SCB patterns based on differential optimization.

According to mentioned above, the specific objectives of this study are as follow: (1) Collecting the original patterns of SCB, and constructing a normalized data set of SCB through data preprocessing and visual analysis. (2) A typical seven layer CNN will be established to implement the automatic recognition and classification of the SCB pattern samples with the Mini-batch Gradient Descent algorithm (MBGD). (3) To shorten the training time, and improve the recognition accuracy of SCB-CNN, the standard differential evolution algorithm will be used to optimize the parameters of SCB-CNN. (4) A SCB-CNN for SCB patterns classification will be established based on differential optimization, which could provide important basic materials for the digital research of bronze patterns and the new ideas for further research on SCB.

## 3 Pattern database and proposed method

**Fig 1:**
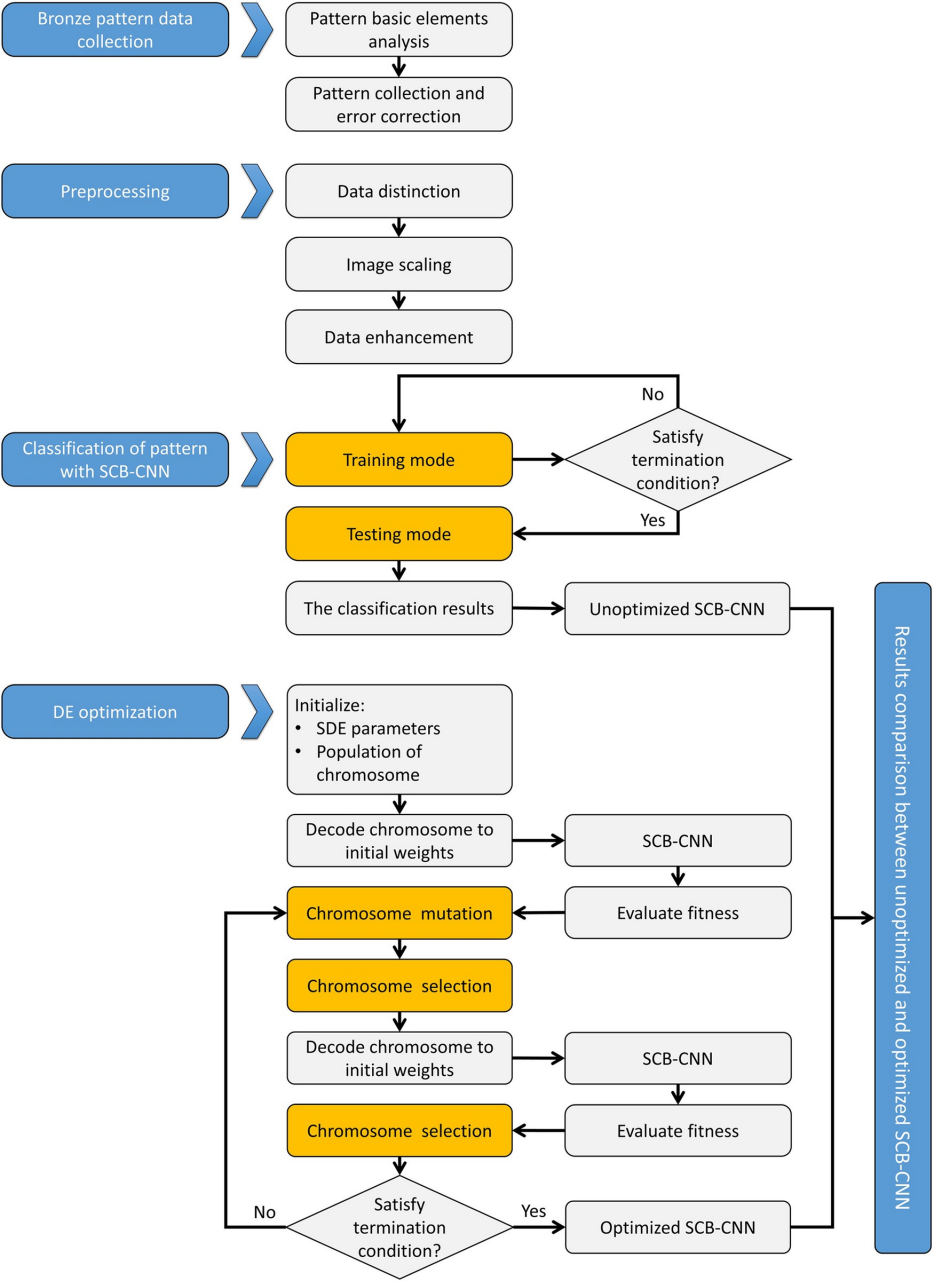
The general framework of evolutionary optimized CNN for bronze patterns.

This section will cover (1) pattern collection and preprocessing, (2) CNN setup, and (3) model optimization. These three aspects introduce the implementation and optimization methods for the pattern classification of SCB. The specific process is depicted in Fig 1.

Fig 1 illustrates the entire process of classifying the patterns of SCB using SCB-CNN. It begins with the collection of bronze patterns from the Shang and Chow Dynasties. After gathering the patterns, we classify and preprocess them to create the bronze pattern database of the Shang and Chow Dynasties. Next, this pattern database is divided into a training dataset and a testing dataset in a 7:3 ratio. SCB-CNN undergoes repeated training to produce pattern classification results and the model itself. Subsequently, the model’s parameters are fine-tuned using the differential evolution (DE) algorithm, resulting in the optimized SCB-CNN.

According to Fig 1, the main pseudocodes designed in this study are as follows, including the primary procedure and DE optimization.

Algorithm 1 is the complete process of the SCB decorative pattern classification algorithm. It achieves automatic classification of decorative patterns on SCB through optimization and evaluation of SCB-CNN, further enhancing classification accuracy and speed. This process includes data preparation, model initialization, parameter optimization, model training, performance evaluation, result analysis, and performance statistics.

The purpose of Algorithm 2 is to optimize the given unoptimized convolutional neural network, which is SCB-CNN, using a differential evolution algorithm to enhance its performance. The primary objective of this algorithm is to discover the optimal combination of parameters that enables SCB-CNN to achieve higher accuracy and efficiency when classifying SCB decorative patterns. It achieves this by generating new parameter sets and employing mutation, crossover, and selection operations to gradually improve and optimize the SCB-CNN model, making it better suited for the specific task. This contributes to improving the success rate of digital inheritance and protection efforts for these cultural heritage artifacts.

Algorithm 1. SCB-CNN Optimization Procedure.

Input: SCB-patterns matrix, alpha, batch size, epochs, population size, generations, F, Cr

Output: Comparison results of unoptimized and optimized SCB-CNN

Load ’SCB’ dataset

Define input_matrix and output_matrix

Split dataset into training and testing sets

Set output activation function as softmax

Set learning rate as alpha

Set batch size as batch size

Set number of training epochs as epochs

Initialize SCB-CNN model

Train SCB-CNN with training data

*# Calculate the total number of parameters to be optimized*

 w1 ← Number of filters in the second convolutional layer * Size of filters in the second convolutional layer

 w2 ← Number of biases in the third layer * Number of filters in the fourth convolutional layer * Size of filters in the fourth convolutional layer

 w_full ← Total number of parameters in the fully connected layers

 sum_para ← w1 + w2 + w_full

*# Optimize SCB-CNN using Differential Evolution (DE)*

 DE_SCB_CNN ← DE_optimization (SCB-CNN, parameters)

 Train the optimized model using training set and optimized parameters

 result ← test (DE_SCB_CNN, test_set)

 Initialize confusion matrix (conf_mat)

 Initialize arrays to keep track of correct predictions

 Initialize arrays to store accuracy for each class

*# Loop through the test data*

 for i  =  1 to amount:

     true_class ← ground_truth (test_set[i])

     predicted_class ← predict (DE_SCB_CNN, test_set[i])

     Update confusion matrix and correct predictions arrays

 Calculate accuracy for each class and store in ACCs array

Algorithm 2. DE optimization for SCB-CNN.

Input: unoptimized CNN: SCB_CNN; DE parameters

Output: The optimized SCB_CNN: DE_SCB_CNN

*# Initialize population*

for i = 1 to population:

   P (i, :) ← InitializeRandomParameters(sum_para)

*# Initialize fitness values*

for i = 1 to population:

   fitness (i), SCB_CNN_fit (i) ← EvaluateFitness (P (i, :), SCB_CNN )

*# Initialize global best individual*

max_value, max_index ← FindBestFitness (fitness)

best_arguments ← P (max_index, :)

best_fitness ← max_value

best_SCB_CNN ← SCB_CNN_fit (max_index)

*# Main DE optimization loop*

for generation = 1 to generations:

   for i = 1 to population:

 *# Mutation*

      V_i,G+1 ← Mutate (P, F, i, generation)      

 *# Crossover*

      U_i,G+1 ← Crossover (P, V_i, i, generation)      

 *# Selection*

      if Fitness (U_i,G+1) < Fitness (P_i,G):

         P_i,G+1 ← U_i,G+1

      else:

         P_i,G+1 ← P_i,G

*# Return the optimized SCB-CNN*

return DE_SCB_CNN

### 3.1 Pattern collection and preprocessing

#### 3.1.1 Pattern collection

Deep learning is an artificial neural network that builds upon the foundation of machine learning by introducing a multi-layer perceptron. Multiple hidden layers constitute the primary characteristic of deep neural networks. Training a deep neural network requires a substantial amount of data. To establish an effective deep learning model, careful consideration must be given to the network structure and the sample data. Data collection and preprocessing play pivotal roles in experiments related to deep learning.

The data to be processed in this study consists of pattern images of SCB. These patterns fall into 3 main categories and 11 sub-categories: animal patterns, geometric patterns, and patterns depicting human activities. The sub-categories include beast faces, dragons, phoenixes, curves, cloud and thunders, strings, fish scales, breasts, dancing, hunting, and war patterns. These decorative patterns are composed of essential elements such as points, lines, and surfaces. These fundamental components shape specific or abstract patterns through various transformations, including changes in size, density, perspective, rotation, regularity, or irregularity. The collection of the aforementioned data was carried out by a specially-assigned individual.

The original images of SCB patterns were sourced from three primary outlets: museums, books, and the internet. The museums involved in this collection effort primarily include the Shaanxi History Museum and the Baoji Bronze Museum. A portion of the data was extracted from relevant books and publications, encompassing academic works on Chinese bronze artifacts, historical literature, and exhibition catalogs. Another portion of the data was obtained from online resources such as The Palace Museum, the National Museum of China, UNESCO, and similar websites, which featured images and information related to bronze artifacts.

**Fig 2:**
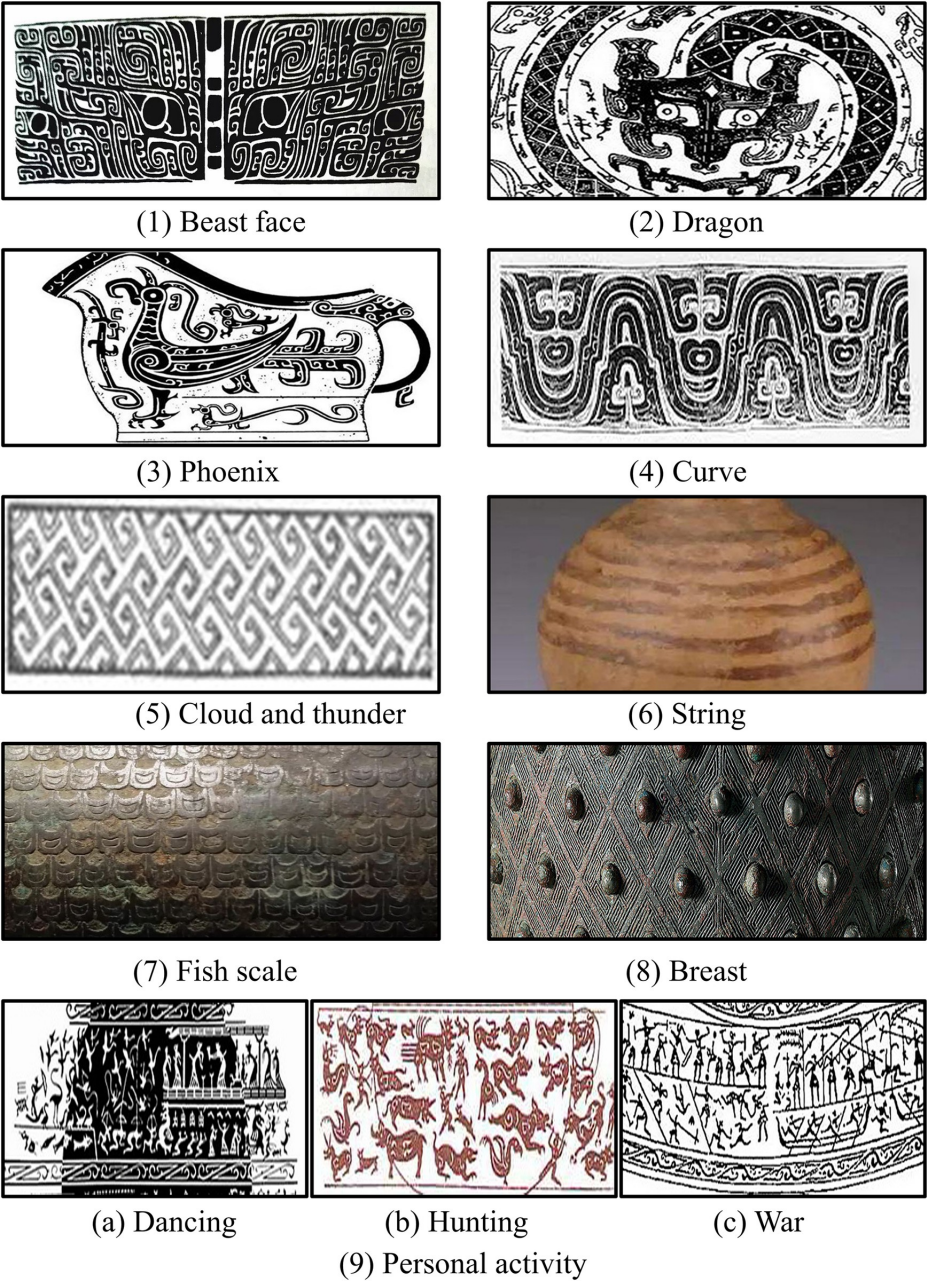
9 types of SCB patterns in the dataset.

Following the completion of data collection, the staff proceeded with the screening and organization of the gathered images to ensure their alignment with the research requirements. Subsequently, dedicated professionals were tasked with assigning categorical labels to each of the collected bronze pattern images, while other team members conducted reviews and corrections to guarantee the accuracy of these label categories. Fig 2 illustrates some samples from the SCB pattern dataset.

The collected data revealed that the textures of the 3 types of personnel activity patterns are complex and highly similar. To minimize the factors that might interfere with model construction and optimization and to simplify model recognition, this study groups dancing, hunting, and war patterns into a single category. As a result, the final classification now comprises a total of 9 categories.

#### 3.1.2 Pattern preprocessing

The collected raw data presented certain imperfections, such as incomplete tables, inconsistent formats, or a lack of features. Data preprocessing comes into play by transforming the original data into a unified format, which is more manageable and addresses the aforementioned issues. Additionally, preprocessing serves the purpose of distinguishing, scaling, and enhancing the images in the database to increase the number of samples and improve network classification accuracy. In this study, preprocessing was applied to the collected SCB pattern images, resulting in a dataset consisting of 9,000 bronze pattern images, with 1,000 images in each of the 9 categories, all standardized in format and size.

Data Splitting: The data is divided into two main parts, namely training data and testing data. When conducting classification training using CNN, it’s essential to differentiate the data in such a way that each training and testing set has a certain proportion of data for all categories. In this paper, the sample data is split in a 7:3 ratio. The training set comprises 7,000 samples of SCB pattern images. These samples are used for model training to learn the recognition and classification of different types of patterns. The testing set consists of 2,000 samples of SCB pattern images. These samples are used to assess the model’s performance and determine whether it can accurately classify new, unseen patterns.

Image Scaling: Images can be resized using a specific scaling factor. Image scaling is a crucial method in pattern preprocessing, and it significantly impacts accuracy. As the input image resolution increases, squared deviations and noise tend to rise, potentially requiring more convolutional and pooling layers for processing. Larger images impose a heavier computational burden, resulting in slower and more complex network training. Conversely, images that are too small may lose important features required for classification and may need to be appropriately enlarged.

Taking into account factors such as training time, widespread use, and scalability of subsequent comparative experiments, this study selected 224x224 as the input image size.

**Fig 3:**
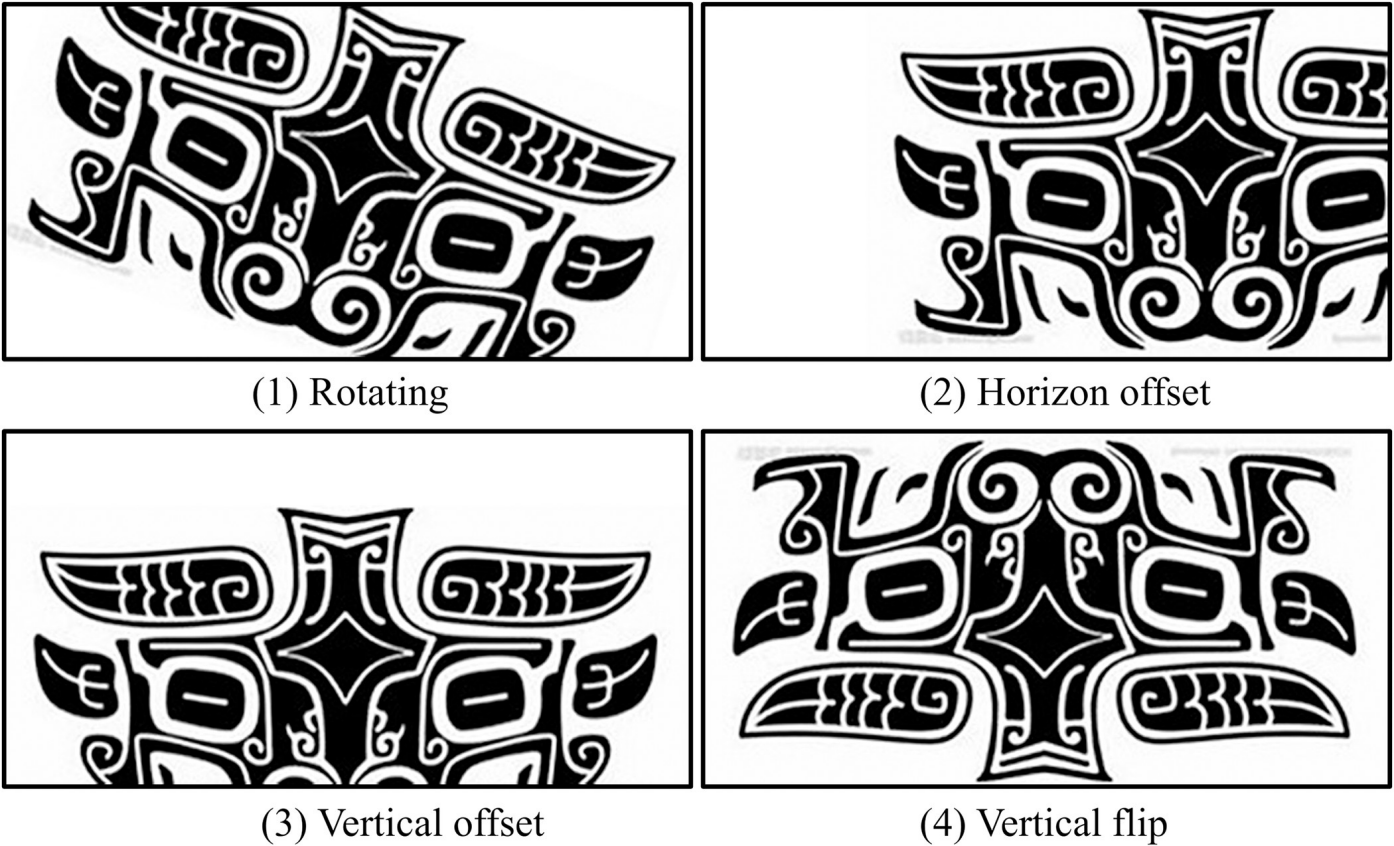
Image preprocessing.

Data Augmentation: When the dataset contains only a small percentage of data, data augmentation can serve as an effective preprocessing method to augment the data samples and meet the requirements of deep learning for large datasets. Data augmentation techniques include horizontal offset, vertical offset, scaling, cropping, zooming, horizontal flipping, vertical flipping, and filling. By expanding the data in this manner, a substantial volume of additional data can be generated. In cases where the training data for a neural network is insufficient, overfitting may occur during the training process, leading to training failure. Even if the network model is successfully trained, it may not perform well on validation data. Utilizing data augmentation techniques helps mitigate overfitting by increasing the dataset’s sample size [36]. Existing datasets are primarily augmented through techniques such as scaling, rotation, cropping, and padding. Fig 3 illustrates four sample images obtained after applying data augmentation to a beast face pattern.

### 3.2 CNN construction

#### 3.2.1 CNN

CNN leverages spatial relationships within the network to reduce the number of parameters that need to be learned, thus enhancing the training performance of the backpropagation (BP) algorithm. A CNN typically comprises an input layer, hidden layers, and an output layer. The hidden layers encompass various convolutional layers, pooling layers, and fully connected layers. The training process of the entire network is divided into two phases: feature forward propagation and error backpropagation. During forward propagation, the current network parameters are used to process the input data, extracting features and ultimately obtaining the classification result. Backpropagation is employed to calculate gradients and propagate errors backward through the network. Finally, the network’s training parameters are updated using the error calculated during the backpropagation phase.

#### 3.2.2 Implementation of SCB-CNN

In this section, this paper delves into the implementation details of SCB-CNN, which plays a crucial role in predicting and classifying 9 different SCB patterns. The SCB-CNN architecture is based on typical convolutional neural networks with a backpropagation training process. This paper breaks down the process into the following key steps:

3.2.2.1 Forward propagation processing

(1) Forward propagation from the input layer to the convolution layer

In this step, the network receives input data of bronze patterns, such as beast face patterns, dragon patterns, and so on. Through convolution operations, the network learns to detect features in the patterns, such as edges, shapes, and *z*^2^more.

The forward propagation of input layer can be expressed as:

$$a_{k}^{2}=f(z^{2})=f\left(\sum_{i=1}^{N}conv2D(a_{i}^{1},w_{ik}^{2})+b_{k}^{2}\right)$$ (1)

The superscript represents the number of layers, *conv*2*D* represents the conventional convolution operation with non-zero boundary filling, *f* is the activation function, *a* is the feature graph, *b* is the bias, *w* is the weight, and *N* is the number of SCB feature graphs.

(2) Forward propagation from the hidden layer to the convolution layer

The hidden layers contain neurons after the convolution layers. In this step, more advanced feature extraction will take place, allowing the network to recognize more complex elements in the patterns.

The main difference between hidden layer and input layer is that the hidden layer’s input is used instead of the matrix produced by the original SCB pattern samples.

$$a_{k}^{l}=f(z^{l})=f\left(\sum_{i=1}^{N}conv2D(a_{i}^{l-1},w_{ik}^{l})+b_{k}^{l}\right)$$ (2)

(3) Forward propagation from the hidden layer to the pooling layer

In this step, the network reduces the data dimensionality through pooling operations while preserving essential information. This helps reduce computational complexity while maintaining bronze patterns’ feature importance. The average pooling is used as the processing logic of the SCB-CNN pooling layer.

(4) Forward propagation from the hidden layer to the fully connected layer

The fully connected layer is used for the final classification decision of 9 kinds of bronze patterns. This step involves comparing the network’s output with the bronze pattern to determine the closest match.

The forward propagation of the hidden layer to the fully connected layer is to put multiple outputs together for calculating the fully connected layer. After calculating the full connection layer, the final output is obtained by the *softmax* activation function.

$$a_{k}^{l}=f(z^{l})=softmax\left(\sum_{i=1}^{N}\left(a_{i}^{l-1}\times w_{ik}^{l}\right)\right)$$ (3)

3.2.2.2 Back propagation processing

Back propagation uses the Mini-batch Gradient Descent (MBGD) to update the weights and offsets of CNN. Because it is necessary to propagate the output error backward step by step, each layer needs to keep the intermediate variables of the error delta (*d*^*l*^) and the derivative (*f*′(*z*^*l*^)) of the activation function.

(1) Error calculation of output layer

In this step, the network calculates the error in the output layer to understand the difference between the model’s prediction and the actual labels, such as determining if a pattern belongs to beast face patterns, which can be expressed as:

$$d^{L}=\frac{\partial C(W,b,x,y)}{\partial a^{L}}={(a}^{L}-y)\times f^{\prime }(a^{L})$$ (4)

Where C is the loss function (cross entropy is used in this case) and *f* is the activation function *softmax* of the output layer.

(2) Error calculation of the last hidden layer

in this step, the network adjusts the weights of the last hidden layer based on backpropagated errors, reducing prediction errors and helping the network capture the features of bronze patterns better, which can be expressed as:

$$d^{l}={\left(W^{l+1}\right)}^{T}\times d^{l+1}\times f^{\prime }(a^{l})$$ (5)

Where *f* is the activation function of the last layer of the hidden layer. Since there is no activation function in the pooling layer, for the sake of the unity of the formula, Let the activation function of the pooling layer be *f*(*z*) = *z*.

(3) Error calculation of other layers in the hidden layer

Similarly, for other hidden layers, errors are calculated and weights are adjusted, further optimizing the network’s performance. If the current layer is a convolution layer and the pooling layer of the upper layer uses average pooling, the error of the current layer is:

$$d^{l}={\left(\frac{\partial a_{k}^{l-1}}{\partial z_{k}^{l-1}}\right)}^{T}\times \frac{\partial f(W,b)}{\partial a_{k}^{l-1}}=upsample(d^{l+1})\times f^{\prime }(a^{l})$$ (6)

Where *f* is the activation function of the current layer, *upsample* needs to complete the logic of pooling error matrix amplification and the error redistribution.

If the current layer is pooled, the error of the current layer is:

$$d^{l}={\left(\frac{\partial z^{l+1}}{\partial z^{l}}\right)}^{T}{\times d}^{l+1}={conv2D(d}^{l+1},rot180(W^{l+1}))\times f^{\prime }(a^{l})$$ (7)

Where *rot*180 is the 180-degree rotation of *W*^*l*+1^ and *conv*2*D* is the conventional convolution operation of zero boundary filling.

3.2.2.3 Update weights and offsets

(1) Hidden layer update

The main task of this step is to use error signals, the network adjusts the weights of the hidden layers to improve the classification accuracy of bronze patterns. The weights and offsets only exist in the convolution layer, so the update here is only for the convolution layer.

$$W^{l}=W^{l}-\alpha \times d_{w}^{l}$$ (8)

$$b^{l}=b^{l}-\alpha \times d_{b}^{l}$$ (9)

Where $d_{w}^{l}$ is the weight error of layer l, $b_{b}^{l}$ is the bias error of layer l, and *α* is the learning rate.

(2) Full connection layer update

Finally, the weights of the fully connected layers are also updated based on error signals, ensuring that the network can better differentiate between different bronze patterns, which can be expressed as:

$$W^{L}=W^{L}-\alpha \times d_{w}^{L}$$ (10)

$$b^{L}=b^{L}-\alpha \times d_{b}^{L}$$ (11)

Where $d_{w}^{l}$ is the weight error of a fully connected layer, $d_{b}^{l}$ is the bias error of the fully connected layer, and *α* is the learning rate.

#### 3.2.3 SCB-CNN structure and parameters

**Fig 4:**
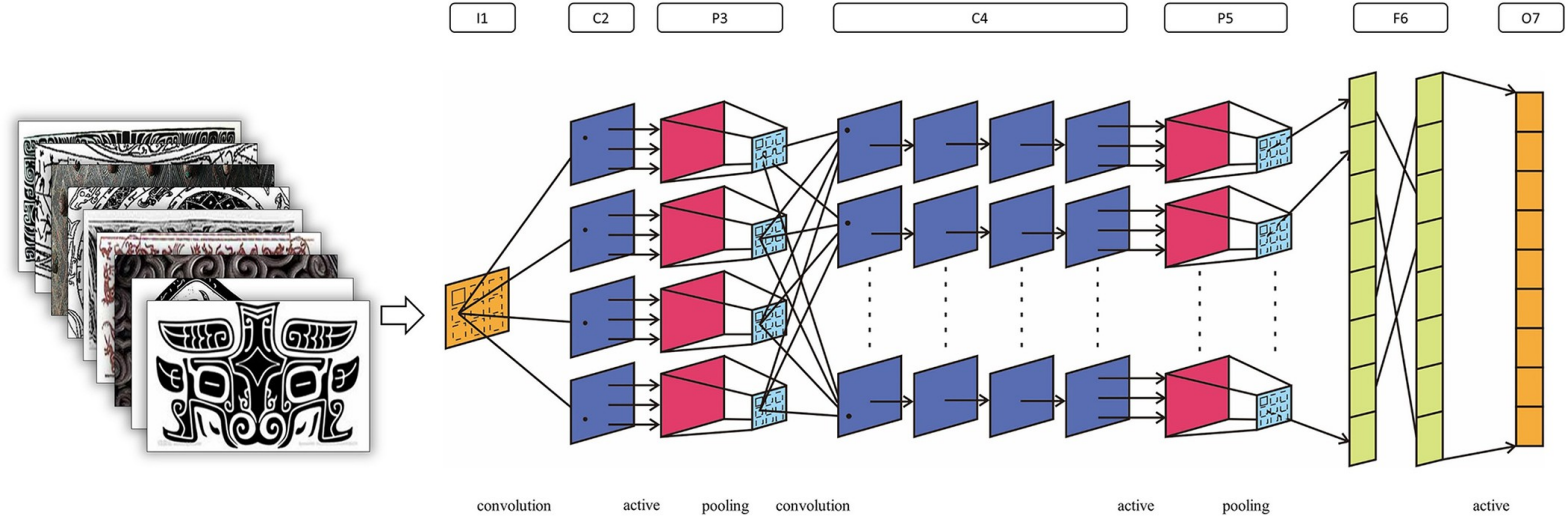
The structure of bronze pattern classification model.

The primary purpose of SCB-CNN is to identify and classify the patterns found on SCB. The SCB-CNN network structure is designed based on the image features of bronze patterns. This design involves adjusting the number of input feature images and selecting the number of hidden layers and pooling combinations. To streamline CNN and its parameters, the convolution and pooling layers are stacked alternately. The network model structure is shown in Fig 4.

**Fig 5:**
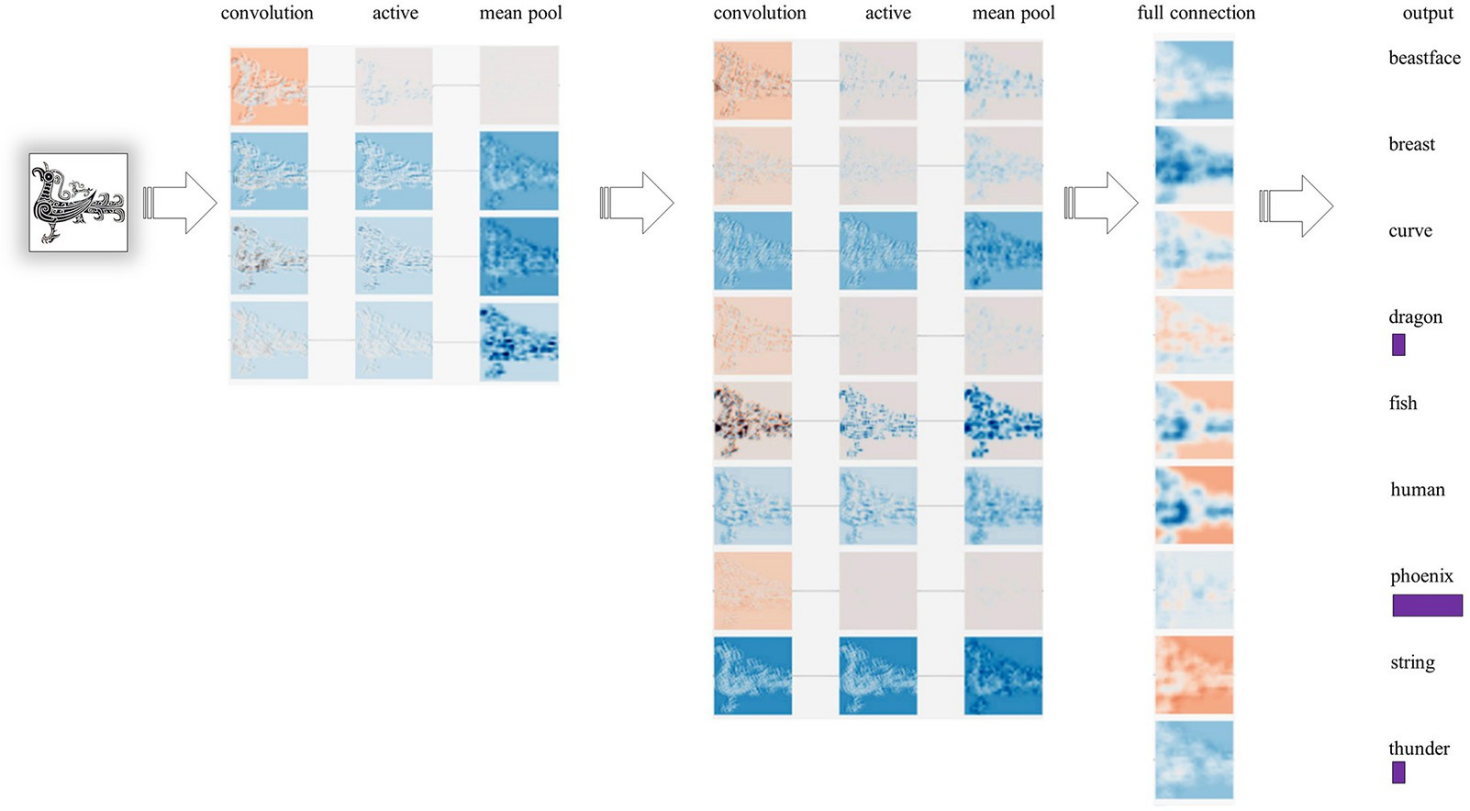
Visual process of feature extraction.

Taking the phoenix pattern as an example, its visual process of feature extraction is shown in Fig 5.

(1) Input layer

For the extracted bronze patterns, although the width and height of each sub-image are equal, the size of each sub-image is not the same. To ensure uniform processing by the model, they must be resized to the same dimensions. In this example, all pattern images are resized to 224x224 pixels.

(2) Convolution layer

The convolution layer extracts image features through convolution operations, with the results serving as parameters for the activation function. As illustrated in Fig 4, C2 and C4 represent convolution layers. The convolution kernel size used in C2 is 7 × 7, with a step size of 1, and zero-padding applied to the width and height. In C4, a 4 × 4 convolution kernel is used with a step size of 1, and zero-padding is applied to the width and height. After processing the input image with 2 convolution layers, we obtain 4 output feature maps of size 218 × 218 pixels and 32 output feature maps of size 106 × 106 pixels, respectively.

(3) Pooling layer

As shown in Fig 4, P3 and P5 represent pooling layers, where each unit in this layer is connected to the neighborhood of the upper layer’s feature map after passing through the activation function with a step size of 0. Both P3 and P5 employ the mean pooling. After two layers of pooling, the output feature maps are sized 109×109 pixels and 53×53 pixels, respectively.

(4) Fully connected layer

F6 represents a fully connected layer. Since the preceding layer yields a total of 8 outputs, each with dimensions of 53 × 53 pixels, and the model comprises a total of 9 categories, the feature vector’s total length in F6 is 202.2K. The classifier calculates the probability for each output category, yielding the final classification result, as depicted in Fig 5. A dropout layer is connected at the output of F6, serving as a regularization method to prevent neural network overfitting and reduce the associated risks. In this study, the dropout is 0.25.

The final parameter design of the SCB-CNN network is shown in Table 1.

**Table 1:** Parameter design of SCB-CNN network.

Type	Parameters	
Input1	size	224×224
Convolution2	output maps	4
	kernelsize	7×7
	activefun	ReLU
Pooling3	scale	2
	mode	Mean
Convolution4	output maps	8
	kernelsize	4×4
	activefun	ReLU
pooling5	scale	2
	mode	mean
Full connection6	activefun	softmax
Output7	lossfun	cross entropy
	dropout	0.25
Learning rate	0.001	

### 3.3 CNN parameters optimization with differential evolution

The Differential Evolution (DE) algorithm is another excellent swarm intelligence optimization algorithm, following the genetic algorithm, particle swarm optimization algorithm, and other evolutionary algorithms [37]. DE features a simple structure, few control parameters, easy implementation of real number coding, fast convergence speed, and its convergence has been theoretically proven [38]. Depending on whether the mutation strategy and control parameters change during the evolution process, the DE algorithm can be divided into the standard Differential Evolution (SDE) algorithm and the adaptive Differential Evolution (ADE) algorithm.

Because the SCB-CNN established in this experiment has demonstrated utility in ECG recognition and classification, considering the algorithm’s complexity and the time required for execution, this section opts for SDE, which has a relatively simple structure, to optimize the initial parameters of SCB-CNN. This process is primarily divided into three stages: SDE optimization implementation, simulation training, and performance evaluation.

The implementation logic of SDE optimization is as follows:

A vector containing *D* optimization variables is called an individual, and the *i*-th individual is represented as:

$$X_{i,G}=[x_{1,i,G}\cdots ,x_{j,i,G}\cdots ,x_{D,i,G}]$$ (12)

Where *i* = 1,2,⋯,*NP*,*NP* represents population size, G represents evolutionary generation, and j represents the *j*-th optimization variable.

**Fig 6:**
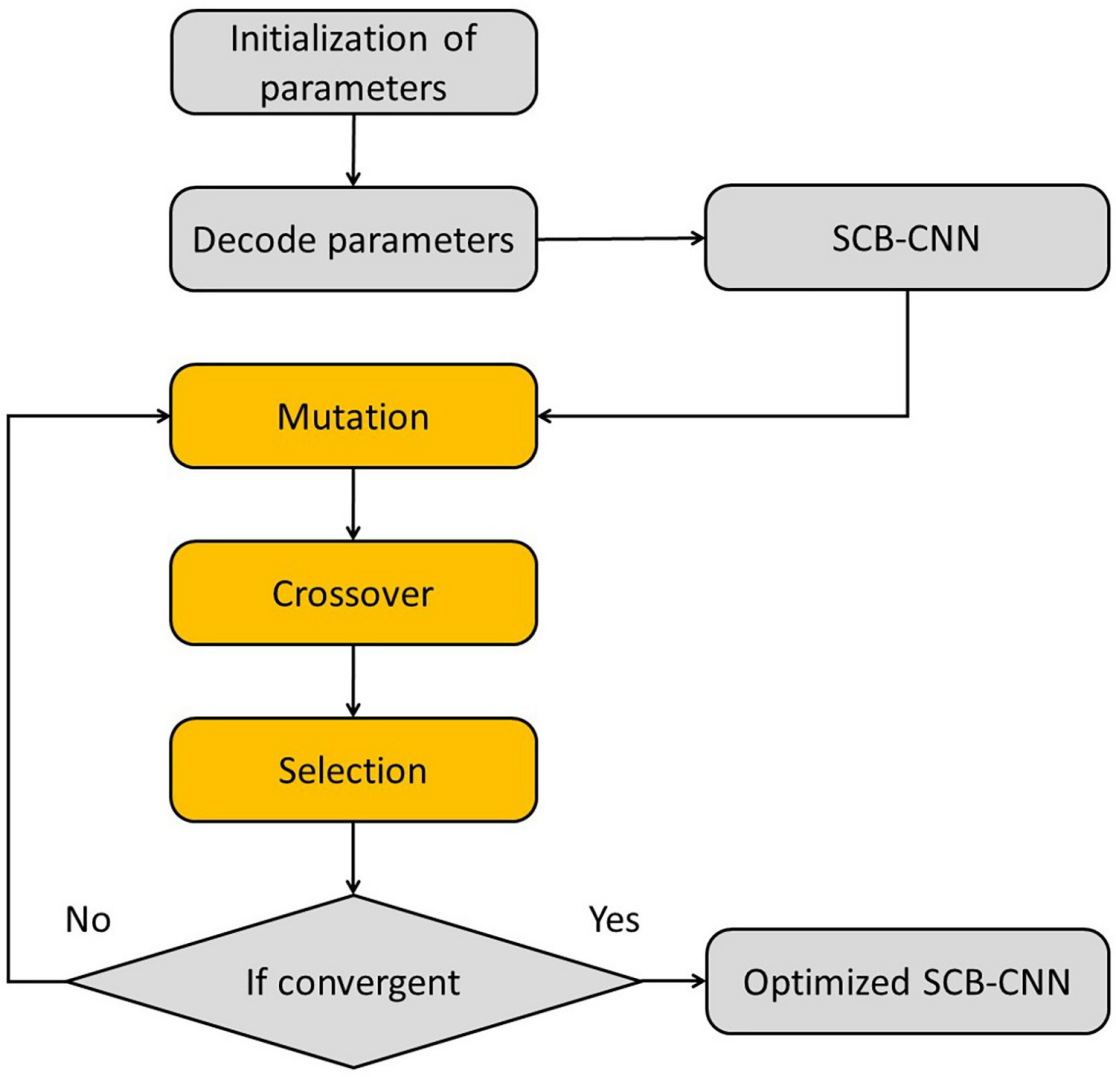
Flow chart of SDE.

The optimization flow chart of SDE for CNN is shown in Fig 6. Its fundamental operations encompass initialization, mutation, crossover, and selection. Initialization primarily involves generating random individuals, while mutation and crossover mainly serve to produce new individuals. The selection operation then determines which individuals will proceed to the next generation. This process is repeated until termination conditions are met.

#### 3.3.1 Mutation

When the population evolves to generation G, the parent individual ***X***_***i*,*G***_ performs a mutation operation to obtain mutant individuals:

$$V_{i,G+1}=X_{r1,G}+F∙(X_{r2,G}-X_{r3,G})$$ (13)

Where the subscripts r1, r2, and r3 are mutually different integers randomly selected between 1 and NP and different from *i*,***X***_***r*1**,***G***_ is called the basis vector, (***X***_***r*2,*G***_−***X***_***r*3,*G***_) is called difference vector and F is mutation operator. If the parameter in the mutated individual exceeds the boundary, the value of the parameter will be replaced by the boundary value.

The mutation strategy used by SDE is usually called DE/rand/1, where "rand" indicates that the basis vector is randomly selected in the population, and "1" indicates the number of difference vectors. DE/rand/1 strategy has good global convergence, but it also has the disadvantage of slow convergence.

#### 3.3.2 Crossover

The test individuals generated by cross operation are as follows:

$$U_{i,G+1}=[u_{1,i,G+1}\cdots ,u_{j,i,G+1}\cdots ,u_{D,i,G+1}]$$ (14)

where,

$$u_{j,i,G+1}=\left\{ \begin{array}{l} v_{j,i,G+1}\;if\;r_{j}[0,1)\leq CR\;or\;j==r(i)\\ x_{j,i,G}\;otherwise \end{array}\right.$$ (15)

Where *r*_*j*_[0,1) represents the random number of the j-th calculation, and CR is the crossover operator. *r*(i) is an integer randomly selected between 1 and D, and ***U***_**i,G+1**_ can obtain at least one variable from ***V***_**i,G+1**_.

#### 3.3.3 Selection

For the minimization problem, in the test individual ***U***_**i,G+1**_ and parent individual ***X***_**i,G**_, select the individual with smaller objective function to enter the next generation population:

$$X_{i,G+1}=\left\{ \begin{array}{l} U_{i,G+1}\;if\;F(U_{i,G+1})<F(X_{i,G})\\ X_{i,G}\;otherwise \end{array}\right.$$ (16)

Where *F*(*X*) represents the objective function.

#### 3.3.4 Control parameters

The control parameters of SDE mainly include population size NP, mutation operator F, and crossover operator CR, which remain unchanged in the process of evolution. The selection of control parameters of SDE follows the empirical rules below.

(1) Population size. According to experience, the population number is usually *NP* = 5*D*∼10*D* to ensure that the algorithm has enough different mutation vectors.

(2) Mutation operator. The value range of mutation operator F is as follows: *F*∈[0,2]. It can be seen from the value range that the mutation operator is a real constant factor, and its size determines the amplification or reduction proportion of the deviation vector. The size of F is negatively correlated with the convergence rate. If the population converges prematurely, F should increase. In this study, the initial value of F is set to 0.5.

(3) Crossover operator. The crossover operator *CR*∈[0,1] is a real constant factor, which is a parameter that controls the probability that the test vector is derived from a randomly selected mutation vector, rather than the original vector.

(4) Maximum evolutionary generation. The general value range is 30~200. According to the needs of the problem, this parameter can also be increased to improve the solution accuracy of the algorithm, but this will make the running time of the algorithm too long.

(5) Termination conditions. The maximum evolutionary generation or fitness threshold can be selected as the termination condition. In this study, the maximum evolutionary generation is selected as the termination condition of optimization.

## 4 Experiments and analysis

In this section, we first construct the SCB-CNN and then implement the SDE optimization for SCB-CNN. Through simulation experiments on the models before and after optimization, we determine the optimal parameter combination. Based on this, the optimized SCB-CNN is compared with the VGG-Net [39] and GoogleNet [40] models to evaluate the performance of the proposed SCB-CNN in the recognition and classification of SCB patterns.

### 4.1 Experimental environment and sample preparation

As shown in Table 2, the experimental operating platform is Windows 10, a 64-bit operating system, with an Intel (R) Core (TM) i7-9850H CPU running at 4.60GHz with 8 processors. The simulation experiments are conducted using Matlab R2017b.

**Table 2:** Software and hardware environment of simulation experiment.

Type	Software environment	Hardware environment
parameter	Windows10, 64bit Matlab R2017b	Intel Core i7-9850H @4.6 GHz, 24GB RAM

For the collected original pattern images, images with obvious defects such as severe distortion, mutual adhesion, shape damage, and low resolution are manually removed. Ultimately, a total of 270 images were obtained for the experiment, comprising 9 categories with 30 images in each category. These categories include beast faces, dragons, phoenixes, curves, cloud and thunders, strings, linked circles, breasts, and personnel activity patterns. Given that the dataset for deep learning networks is not sufficiently large, data augmentation techniques were employed to expand the number of samples. Data augmentation operations included rotating the image at various angles, vertical and horizontal mirroring, displacement up and down, cropping, and more. Through data augmentation, a dataset of 1000 patterns for each type was created, resulting in a total of 9000 patterns. The ratio of pattern images in the training set to the testing set is 7:3, with 7000 patterns used for the training set and 2000 patterns used for testing.

### 4.2 Classification training process and analysis

Given the aforementioned software and hardware environment, the hyperparameter settings for this model are as follows. The initial learning rate is set to 0.001. Typically, smaller learning rates require more epochs to converge, while larger learning rates allow for quicker weight updates. The model utilizes mini-batch stochastic gradient descent for training. To ensure better gradient estimation, each batch contains multiple samples, and the batch size for the training data is set to 16. During the training process, in each training epoch, 16 images are randomly selected, and each image is used multiple times. Additionally, 16 images are chosen for cross-validation, and precision (P+) is calculated once per training epoch, followed by an evaluation of the training results. To ensure there is enough time for training and convergence, this model sets the number of epochs to 100. The above hyperparameter settings will be further evaluated through subsequent ablation experiments.

**Fig 7:**
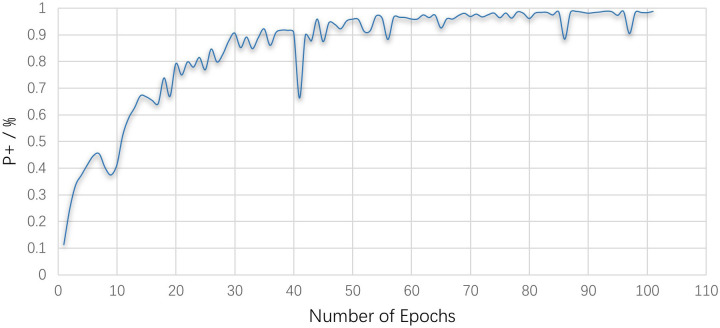
The positive prediction rate during model training.

**Fig 8:**
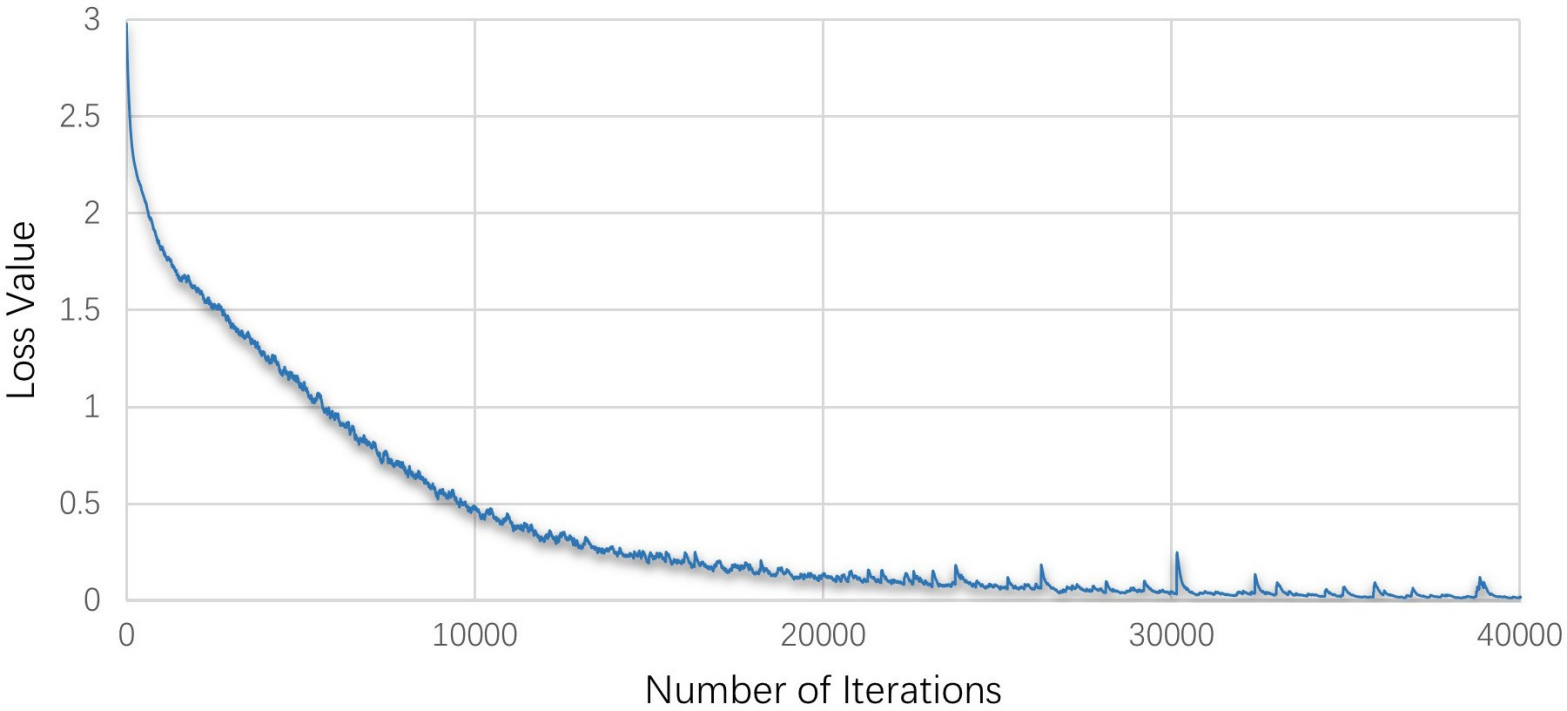
The loss value during model training.

The change process of P+ and loss value with epoch and iteration in the training process of this experiment is shown in Figs 7 and 8.

The loss value during the training process represents the error between the model’s predicted results for the training set and the actual results, while P+ during the training process denotes the percentage of randomly selected images that are correctly classified. In the training process, a smaller loss value indicates better learning performance, and a higher P+ corresponds to greater accuracy. After each training iteration, the predicted values are compared to the actual values and used in backpropagation to adjust the weights of each network layer, optimizing network parameters and enhancing network performance. Figs 7 and 8 show that the classification accuracy of the testing set continues to improve during the testing process, while the training loss value consistently decreases. After a certain number of epochs and iterations, the network’s performance stabilizes. During the first 30 epochs of training, the test accuracy rate increases rapidly, and after 50 epochs, the accuracy rate increases more gradually, ultimately stabilizing at over 95%. As for the training loss value, it decreases rapidly in the first 10,000 iterations, followed by a slower decrease and stabilization around 0.03. After 30,000 iterations, the model converges to its optimal state.

To prevent the influence of contingency on the results, this study uses the average accuracy to evaluate the classification performance of the algorithm. Based on maintaining the network structure parameters and training initial parameters, 30 independent experiments are carried out on the network model. It is observed that the test classification accuracy is (98.8 ± 0.05)%, indicating that this model can effectively suppress over-fitting while obtaining high test classification accuracy. According to the changes in training loss value and test accuracy, it can be seen that the training effect of the network model is ideal. The proposed SCB-CNN model can accurately classify bronze patterns.

While SCB-CNN exhibits high classification accuracy, it requires a significant amount of training time. As depicted in Fig 8, the model training involved a total of 43,800 iterations. As the resolution of pattern images increases, training time sharply rises. Therefore, in the next step, we will employ SDE to optimize the initial parameters of SCB-CNN, aiming to effectively reduce the training time of SCB-CNN while maintaining high classification accuracy.

### 4.3 SDE optimization simulation and analysis

#### 4.3.1 Simulation process

**Fig 9:**
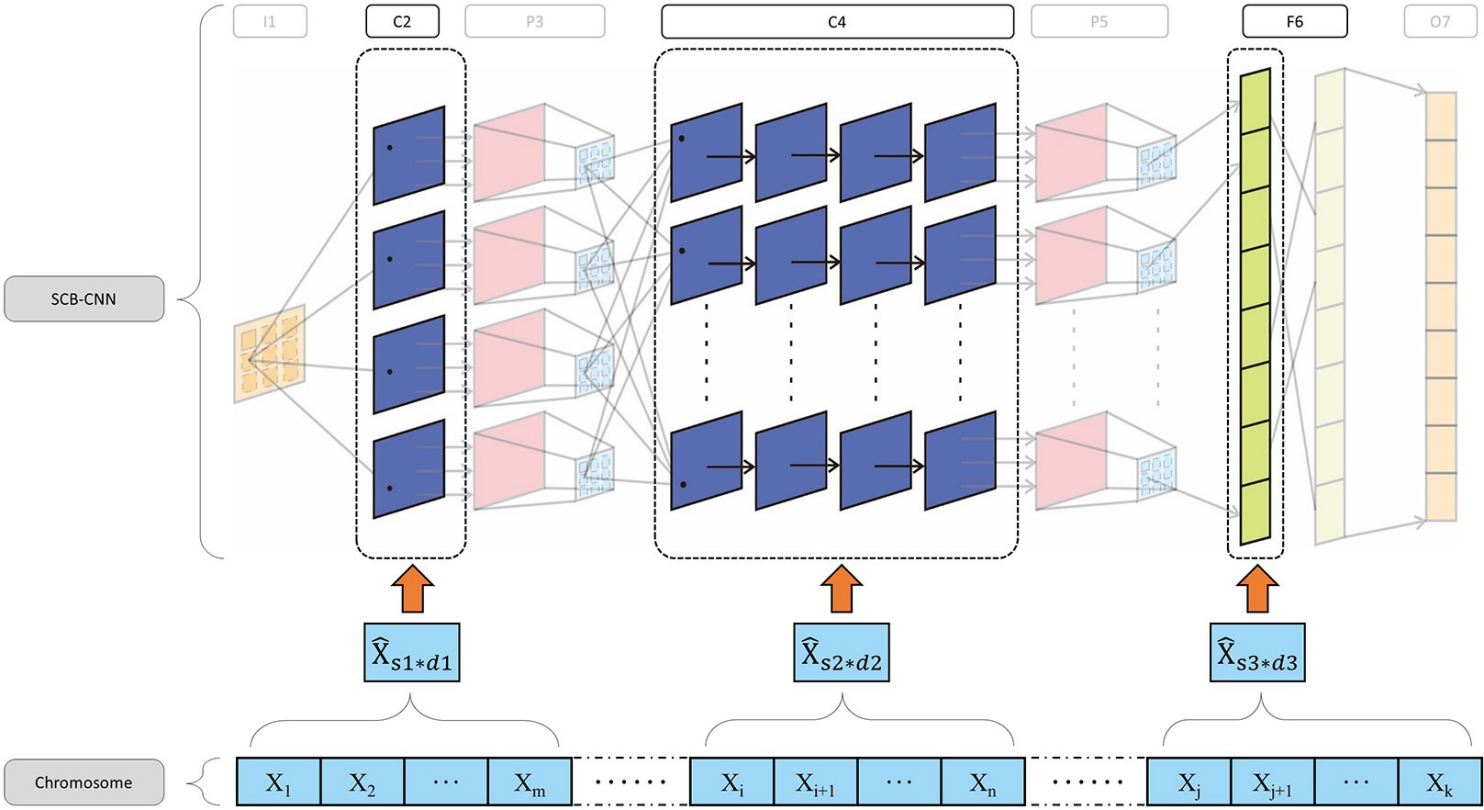
The mapping from chromosome to weights.

The simulation process of SDE optimization is conducted following the procedure outlined in Section 2.3. The software and hardware environment for the simulation experiment remains the same as described in Section 3.1. The optimization target is the SCB-CNN model established in Section 3.2, including its initial parameters. The dataset employed by SCB-CNN still consists of 9 types of bronze patterns and their respective classification labels.

experiment process:

(1) Import and establish a total of 9000 pattern datasets and corresponding label sets.

(2) Normalize the dataset to remove the influence of the baseline.

(3) According to the ratio of 7:3, the data set is randomly divided into 7000 training set and 2000 testing set, and each type has a data volume of 1000 pattern images.

(4) According to the parameter design in 2.2, establish and initialize SCB-CNN and initialize the parameters of SDE. Set the population size to 30 and the maximum evolutionary generation to 50, the initial value of mutation operator to 0.5, and the initial value of crossover operator to 0.9.

(5) Calculate the total number of parameters to be optimized. This data is usually called chromosome and generates a parameter vector with a length of 202.2K.

(6) According to the data in step 5, initialize the population element to a random number between (-1,1) and generate a population random matrix with a size of [30, 202.2K].

(7) Split the parameter vector in DE into the organizational structure of the corresponding weight in SCB-CNN, assign it to the weight matrix, and initialize the corresponding fitness at the same time. The structural mapping of chromosomes to weights is shown in Fig 9. Among them, ${\hat{X}}_{s*d}$ represents the mapping of chromosomes to weights, the subscript s represents the dimension of the weight, and d represents the dimension of the weight.

(8) Calculate the optimal fitness and optimal parameter vector of the population in every iteration through mutation, crossover, and selection. The fitness function adopts the P+ of SCB-CNN.

(9) Repeat step 7 until the iteration is complete. Record the final fitness and parameter vector, split the parameter vector and assign it to SCB-CNN, and the process of optimization is over.

#### 4.3.2 Performance analysis

As the chosen fitness function is the positive prediction rate of SCB-CNN, algorithm performance can be enhanced through an analysis of the fitness function. To generalize the problem, during the optimization process, parameters other than the mutation operator F and crossover operator CR are not adjusted.

DE determines algorithm termination based on a maximum evolutionary generation or a threshold. In this study, the maximum evolutionary generation is chosen as the termination condition for DE, with the final P+ of the SCB classification serving as the reference threshold.

The typical range for the maximum evolutionary generation is usually between 50 and 200. Depending on the problem’s requirements, increasing the evolutionary generation can enhance the algorithm’s solution accuracy, but it can also significantly extend the algorithm’s runtime. Conversely, while maintaining solution accuracy, reducing the generation can shorten the algorithm’s runtime [41]. Additionally, from experiments involving the optimization of multivariate functions, it has been observed that the algorithm converges effectively with a generation count of no less than 30. Therefore, in this study, we attempt to set the maximum evolutionary generation to 30 in the hope of further shortening the convergence time of the algorithm, while ensuring high classification accuracy and maintaining the experiment’s objectivity through comparative analysis.

As mentioned above, the mutation operator determines the amplification ratio of the deviation vector, and its size is negatively correlated with the convergence speed. On the other hand, the crossover operator is controlled by experimentally selected vectors, and the size of the crossover operator is positively correlated with the convergence rate. Previous studies [42] have shown that mutation operators less than 0.4 and greater than 1.2 are only occasionally effective. Furthermore, literature [43] has also pointed out that the loss of individual diversity within the population is the primary cause of premature population convergence. Therefore, to ensure population diversity and improve search capabilities [44], it is crucial to control the appropriateness of parameter settings, particularly regarding the contraction factor F and crossover factor CR. In the experiments, we initially set F = 0.5 and CR = 0.9 as the starting values. We then varied F between 0.5, 0.8, 1.0, and 1.2, and CR between 0.9 and 0.8 as parameter combinations to systematically test the optimization results of the algorithm step by step.

Since the final evaluation criterion is the P+ result of SCB-CNN, similar to the previous discussion, to mitigate the impact of classification contingencies on result analysis, we continue to employ average accuracy to assess the algorithm’s classification performance. We conducted 30 independent experiments on the classification algorithms using different DE parameter combinations, with each experiment comprising 20 epochs. The final accuracy is calculated as the average of the 30 classification accuracy scores. The optimization results are presented in Table 3, which reflects the average outcomes from these 30 repeated experiments.

**Table 3:** Statistics of DE optimization results.

	DE phase				CNN phase	
	**Group**	**F**	**CR**	**P+**	**SCB-CNN epochs**	**P+**
ReLU	**1**	**0.5**	**0.9**	**0.756**	**20**	**0.988**
	2	0.8	0.9	0.745	20	0.978
	3	1	0.8	0.628	20	0.967
	4	1.2	0.8	0.668	20	0.975

**Fig 10:**
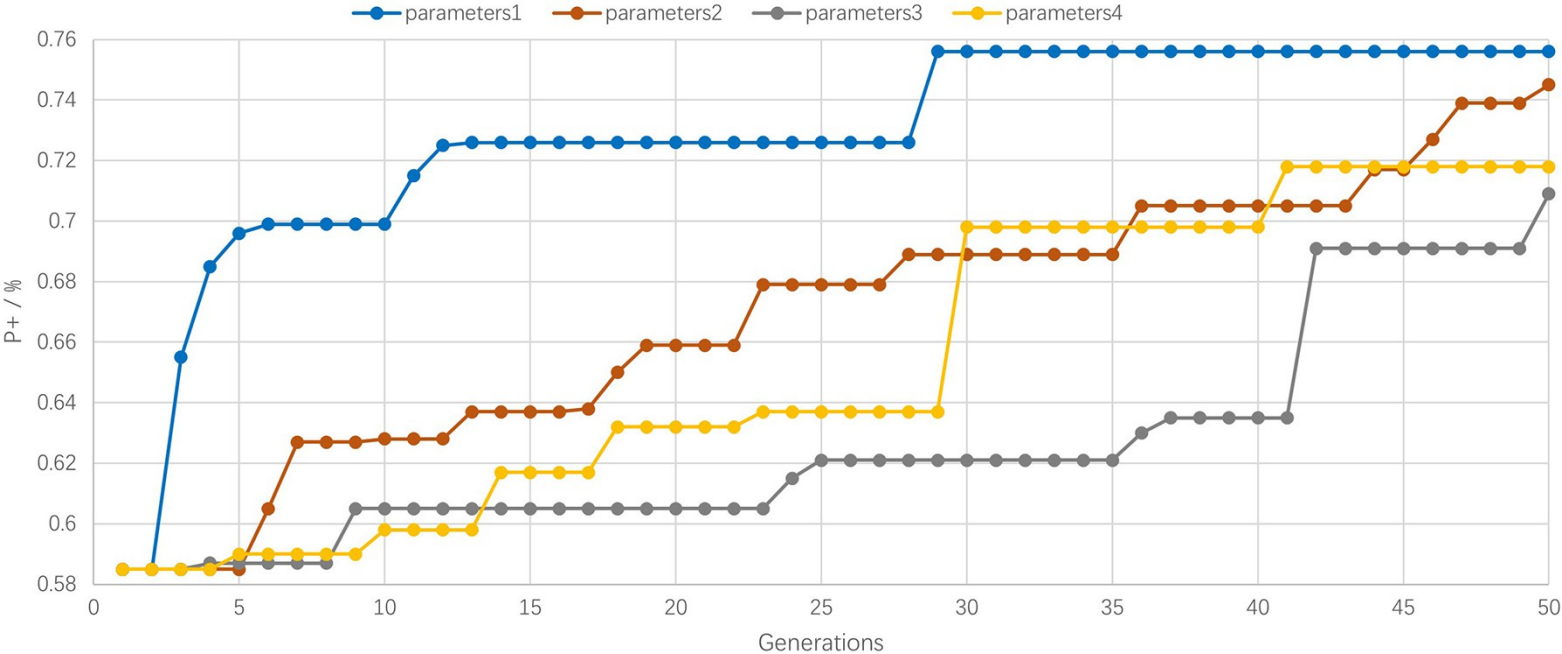
ReLU fitness function line graph.

Fig 10 shows the fitness function graph of different DE parameter combinations with ReLU as the activation function.

#### 4.3.3 Ablation experiments and analysis of optimized SCB-CNN

To gain a deeper understanding of the characteristics and behavior of SCB-CNN, optimize model performance, and provide more targeted recommendations for future model improvements, this section conducts ablation experiments on the optimized SCB-CNN. The primary factors under investigation include convolution layers, pooling layers, data augmentation, learning rate, batch size, and the number of epochs. With the exception of the number of epochs, which is based on the average of five independent random experiments, the data for the other factors are derived from the averages of 30 independent random trials, as shown in Table 4.

**Table 4:** SCB-CNN ablation experiment results.

Experimental Conditions			Data Augmentation	Training Time (S)	P+
optimized SCB-CNN	learning Rate	0.001	Enable	262.7	0.988
	batch	16			
	epochs	20			
removing a Convolution layer			Enable	142.1	0.620
removing a Pooling layer			Enable	207.4	0.876
disabling data augmentation			Disable	202.3	0.774
removing Dropout layer			Enable	220.7	0.905
learning Rate		0.01	Enable	157.6	0.966
		0.0001	Enable	446.6	0.988
batch		8	Enable	281.1	0.971
		32	Enable	239.1	0.954
epochs		200	Enable	3231.2	0.989

Based on the experimental results, it can be observed that the factors with the most significant impact on model training time are the number of epochs, convolution layers, and learning rate. The factors with the most substantial impact on model accuracy are convolution layers, pooling layers, and data augmentation. When the epoch is set to 200, the average training time increases to 3231.2 seconds, resulting in an accuracy improvement to 0.989. Removing one convolution layer reduces the average training time to 142.1 seconds but lowers accuracy to 0.62. Setting the learning rate to 0.0001 increases the average training time to 446.6 seconds while maintaining the same accuracy. Eliminating one pooling layer reduces the average training time to 207.4 seconds but reduces accuracy to 0.876. Removing data augmentation reduces the average training time to 202.3 seconds but decreases accuracy to 0.774.

The above results indicate that SCB-CNN relies heavily on convolution and pooling layers, and these components are crucial for the model’s performance, making them indispensable. Additionally, the data augmentation provided in this study significantly enhances the model’s ability to generalize, exerting a tangible impact on the model’s performance. Considering the trade-off between time and P+, setting the learning rate to 0.001, the batch size to 16, and the epoch to 20 appears to be a reasonable choice. These parameters will also be employed in subsequent comparative experiments.

#### 4.4 Comparative experiments before and after optimization

This study uses the confusion matrix to represent the classification results. in SCB pattern Database, the feature data sets of 9 types of pattern are obtained, of which 1000 are selected for each type, and the total data is 9000. 7000 of them are assigned to the training set and 2000 to the testing set. Table 5 shows the confusion matrix results of the testing data set from SCB pattern Database under ReLU function, which is the average of 30 repeated experiments.

**Table 5:** Confusion matrix of 2000 sample test set with ReLU.

**Actual**		**Prediction**									
		beastface	breast	curve	dragon	fish	human	phoenix	string	thunder	P+
	beastface	158	2	5	11	16	0	7	0	31	68.7%
	breast	1	201	0	0	1	0	3	2	30	84.5%
	curve	0	7	146	2	27	0	11	15	7	67.9%
	dragon	**5**	**7**	**7**	**133**	**5**	**3**	**6**	**6**	**47**	**60.7%**
	fish	6	0	3	0	162	0	5	18	19	76.1%
	human	**3**	**0**	**0**	**3**	**0**	**229**	**1**	**0**	**0**	**97.0%**
	phoenix	1	0	1	5	3	0	216	0	9	91.9%
	string	0	0	0	0	3	0	0	201	14	92.2%
	thunder	0	7	0	0	0	0	0	17	172	87.8%
	Total	171	226	159	151	217	232	250	265	329	**80.8%**

The diagonal data in the confusion matrix is the result of correct classification. Among them, the **human** type has the highest accuracy, reaching 97%, and the **dragon** type has the lowest accuracy, only 60.7%. From the data in the Table above, we can find that without DE optimization, the average classification accuracy after 20 epochs is 80.8% with ReLU. The apparent difference in accuracy is due to the quality of sampling patterns, and the degree of homogeneity within the same type of samples.

According to the statistics of DE optimization results in Table 3, the best combination of DE parameters is selected, and the optimization comparison experiment is carried out on SCB-CNN. The experiment results are shown in Table 6.

**Table 6:** P+ and Time comparison of different stages before and after DE.

Measure	Active function	Before Optimization					After Optimization			
		initial	10 epochs	20 epochs	50 epochs	100 epochs	Generation	initial	10 epochs	20 epochs
*P+*	ReLU	0.151	0.646	**0.808**	0.968	**0.979**	50	0.756	0.974	**0.988**
*Time/S*		--	124.53	**261.44**	618.69	**1354.48**		--	122.32	**262.67**

It can be seen from Table 6 that after 50 generations of DE optimization and 20 training cycles of SCB-CNN epochs, its accuracy reaches or exceeds the 100 training effects of the non-optimized SCB-CNN. The reason is that the initial parameters of the optimized SCB-CNN have structural characteristics. Because the data set is trained with structured parameters, the gradient descent algorithm in the BP process will make the loss function converge faster, and finally, make the model ensure high recognition accuracy while reducing the number of training.

It can also be seen from this Table that under the condition that the total number of 9000 samples, the comparison results of the time consumed in different epoch stages of SCB-CNN before and after optimization, which is also the average value after 30 independent experiments.

As mentioned above, the accuracy of the optimized SCB-CNN reached or exceeded the accuracy achieved by the unoptimized SCB-CNN after 20 epochs. Therefore, our primary focus is on comparing the time consumed to achieve these accuracy levels. From the bold column in Table 6, it’s evident that there is a significant reduction in time consumption, and this reduction becomes even more pronounced with increasing model complexity and the total number of samples. In other words, the optimization effect of DE becomes more pronounced when dealing with more complex models and larger sample sizes.

It can be seen from Table 7 that the different combinations of the variation factor F and the cross factor CR determine the convergence speed and optimization effect in the DE stage. The F value is negatively correlated with the convergence, and the CR value is positively correlated with the convergence rate. However, the best SCB-CNN classification accuracy does not necessarily come from the best optimization results in the DE stage, because the optimization strategies adopted by the two algorithms are different. DE uses a stochastic optimization method based on genetic algorithm, and SCB-CNN uses a gradual optimization method based on gradient descent.

**Table 7:** Comparisons of P+ with different configurations of SDE and SCB-CNN.

Active function	epoch	Time/S	Before optimization	SDE optimization						
				*F*	*CR*	generation	Initial P+	epoch	Final P+	Time/S
ReLU	20	261.4±5	0.808	0.5	0.9	50	0.756	20	0.988	262.7±5
				0.8	0.9		0.745		0.978	
				1	0.8		0.628		0.967	
				1.2	0.8		0.668		0.975	
	100	1354.5±5	0.979	0.5	0.9	50	0.756	100	0.991	1366.27±5
				0.8	0.9		0.745		0.985	
				1	0.8		0.628		0.972	
				1.2	0.8		0.668		0.981	

### 4.5 Comparative experiments with other models

In this section, the proposed SCB-CNN after optimization is evaluated for its performance in SCB patterns detection and classification by comparing it with VGG16 and GoogleNet. The comparison criteria include prediction speed, convergence speed, precision, and training time. Prediction speed refers to the number of images processed by the model per second, convergence speed denotes the number of epochs required for the model to reach convergence during training, and precision is calculated using the formula:

$$P+=\frac{TP}{TP+FP}$$ (17)

TP: True Positives,

FP: False Positives

To reduce the impact of randomness on the experiments, the following data are all based on the averages of 30 independent experiments.

#### 4.5.1 Comparison of the proposed SCB-CNN with VGG-Net

VGG-Net is a deep CNN architecture developed by the research team at the University of Oxford. Common VGG-Net architectures include VGG-16 and VGG-19. Compared to VGG-19, VGG-16 has a simpler structure, requires less training time and computational resources. Moreover, since VGG-16 has fewer differences compared to the optimized SCB-CNN, experiments comparing the two models can more clearly demonstrate the optimization effect. Therefore, in this experiment, VGG-16 is chosen as the comparative model. The parameters of VGG-16 are shown in the Table 8 below, and the model takes fixed-size 224x224 gray bronze patterns as input.

**Table 8:** Configuration of the VGG-16.

No.	Layer	Type	Output	numbers	Stride	Padding	Other Parameters
1	Imput	-	224x224x1	-	-	-	-
2	Convolution 1–1	Convolution	224x224x64	0.6K	1	1	3x3, ReLU
3	Convolution 1–2	Convolution	224x224x64	36.9K	1	1	3x3, ReLU
4	Max Pooling 1	Max Pooling	112x112x64	-	2	0	2x2
5	Convolution 2–1	Convolution	112x112x128	73.7K	1	1	3x3, ReLU
6	Convolution 2–2	Convolution	112x112x128	147.5K	1	1	3x3, ReLU
7	Max Pooling 2	Max Pooling	56x56x128	-	2	0	2x2
8	Convolution 3–1	Convolution	56x56x256	294.5K	1	1	3x3, ReLU
9	Convolution 3–2	Convolution	56x56x256	589.5K	1	1	3x3, ReLU
10	Convolution 3–3	Convolution	56x56x256	589.5K	1	1	3x3, ReLU
11	Max Pooling 3	Max Pooling	28x28x256	-	2	0	2x2
12	Convolution 4–1	Convolution	28x28x512	1.1M	1	1	3x3, ReLU
13	Convolution 4–2	Convolution	28x28x512	2.3M	1	1	3x3, ReLU
14	Convolution 4–3	Convolution	28x28x512	2.3M	1	1	3x3, ReLU
15	Max Pooling 4	Max Pooling	14x14x512	-	2	0	2x2
16	Convolution 5–1	Convolution	14x14x512	2.3M	1	1	3x3, ReLU
17	Convolution 5–2	Convolution	14x14x512	2.3M	1	1	3x3, ReLU
18	Convolution 5–3	Convolution	14x14x512	2.3M	1	1	3x3, ReLU
19	Max Pooling 5	Max Pooling	7x7x512	-	2	0	2x2
20	Full connection 1	Full connection	4096	102M	-	-	ReLU, Dropout 0.5
21	Full connection 2	Full connection	4096	16.2M	-	-	ReLU, Dropout 0.5
22	Full connection 3	Full connection	9	36.1K	-	-	categories:9, No activation, No Dropout
23	Output	Full connection	9	87	-	-	Softmax

The optimized SCB-CNN uses the same model structure as described in Section 4.4. Both models have a learning rate of 0.001, and the batch size is set to 16. The database contains a total of 9 classes of SCB patterns, with a total of 9,000 data samples. Among these, 7,000 samples are allocated to the training set, and 2,000 samples are allocated to the test set.

Table 9 is the prediction speed comparison between the two models, Table 10 is the convergence speed comparison between the two models, and Table 11 is the final classification performance comparison between the two models. The results indicate that after multiple runs, the data for VGG-16 is more concentrated, resulting in faster prediction speed; SCB-CNN exhibits a faster convergence speed as it requires fewer epochs to reach convergence compared to VGG-16; Although both models achieve high P+ after the same number of epochs, VGG-16 requires more training time.

**Table 9:** Comparison of prediction speed.

Model	Average Speed (samples/S)	Max Speed (samples/S)	Min Speed (samples/S)	SD (samples/S)	Prediction Speed (samples/S)
**Optimized SCB-CNN**	66	74	64	4	69
**VGG-Net**	60	66	56	2	61

**Table 10:** Comparison of convergence speed.

Model	Performance Improvement at Fast Convergence (%)	Convergence Stability (%)			Epochs
		Average Performance	SD	Moving Average	
**Optimized SCB-CNN**	25	0.92	0.03	0.93	5
**VGG-Net**	20	0.88	0.05	0.89	15

**Table 11:** The classification performance of SCB-CNN and VGG-16.

Measure	SCB-CNN				VGG-16			
	10 epochs	20 epochs	50 epochs	100 epochs	10 epochs	20 epochs	50 epochs	100 epochs
**prediction speed**	69	69	69	69	61	61	61	61
**convergence speed**	5	5	5	5	15	15	15	15
***P+***	0.974	0.988	0.989	0.991	0.687	0.835	0.976	0.988
***Time/S***	122.32	262.67	633.62	1366.27	236.37	481.92	1204.85	2554.76

### 4.5.2 Comparison of the proposed SCB-CNN with GoogleNet

To further investigate the performance of the proposed SCB-CNN, it is compared with GoogleNet in the classification of SCB patterns. SCB-CNN’s configuration remains consistent with Section 4.4. GoogleNet (Inception-v1) has a highly complex network structure, including multiple Inception modules and various types of layers. Table 12 shows the key layers used in this experiment with their corresponding parameter settings for GoogleNet. Except for the last layer, all weight layers use the ReLU activation function. The models take fixed-size 224×224 gray SCB pattern images as input. Both models use a learning rate of 0.001, zero-mean unit-variance for data normalization, and have a batch size of 16.

**Table 12:** Configuration of the GoogleNet model.

No.	Layer	Type	Output	stride	padding	Other Parameters
1	Imput	-	224x224x1	-	-	-
2	Convolution1	Convolution	112x112x64	2	1	7x7, ReLU
3	Max Pooling 1	Max Pooling	56x56x64	2	0	3x3,
4	Convolution2	Convolution	56x56x192	1	1	3x3, ReLU
5	Max Pooling 2	Max Pooling	28x28x192	2	0	3x3,
6	Inception 1	Multi-Branch Convolution	28x28x256	-	-	-
7	Inception 2	Multi-Branch Convolution	28x28x480	-	-	-
8	Max Pooling 3	Max Pooling	14x14x480	2	0	3x3,
9	Inception 3	Multi-Branch Convolution	14x14x512	-	-	-
10	Inception 4	Multi-Branch Convolution	14x14x512	-	-	-
11	Inception 5	Multi-Branch Convolution	14x14x512	-	-	-
12	Max Pooling 4	Max Pooling	7x7x512	2	0	3x3,
13	Global Average Pooling	Average Pooling	1x1x512	-	-	Global Average Pooling
14	Full connection 1	Full connection	4096	-	-	ReLU, Dropout 0.5
15	Full connection 2	Full connection	4096	-	-	ReLU, Dropout 0.5
16	Full connection 3	Full connection	9	-	-	categories:9
17	Output	Full connection	9	-	-	Softmax

Table 13 shows the comparison of prediction speeds between the two models, Table 14 presents the comparison of convergence speeds between the two models, and Table 15 displays the comparison of the final classification performance between the two models. The results indicate that after multiple runs, the data for GoogleNet is more concentrated, resulting in faster prediction speeds compared to SCB-CNN. SCB-CNN exhibits a faster convergence speed as it requires fewer epochs to reach convergence compared to GoogleNet. Although after 100 epochs, GoogleNet achieves a slightly higher P+ compared to SCB-CNN, SCB-CNN demonstrates significantly shorter training times at each stage.

**Table 13:** Comparison of prediction speed.

Model	Average Speed (samples/S)	Max Speed (samples/S)	Min Speed (samples/S)	SD (samples/S)	Prediction Speed (samples/S)
**Optimized SCB-CNN**	66	74	64	4	69
**GoogleNet**	70	75	65	2	70

**Table 14:** Comparison of convergence speed.

Model	Performance Improvement at Fast Convergence (%)	Convergence Stability (%)			Epochs
		Average Performance	SD	Moving Average	
**Optimized SCB-CNN**	25	0.92	0.03	0.93	5
**GoogleNet**	22	0.87	0.06	0.88	15

**Table 15:** The classification performance of SCB-CNN and GoogleNet.

Measure	SCB-CNN				GoogleNet			
	10 epochs	20 epochs	50 epochs	100 epochs	10 epochs	20 epochs	50 epochs	100 epochs
**prediction speed**	69	69	69	69	70	70	70	70
**convergence speed**	5	5	5	5	15	15	15	15
***P+***	0.974	0.988	0.989	0.991	0.692	0.898	0.983	0.993
***Time/S***	122.32	262.67	633.62	1366.27	322.51	657.31	1639.72	3293.37

Table 16 integrates the classification performance of all models. The results reveal that GoogleNet has the fastest prediction speed, with the optimized SCB-CNN closely following, followed by VGG-Net, and the unoptimized SCB-CNN being the slowest; After 100 epochs, GoogleNet achieves the highest accuracy, reaching 0.993, followed by the optimized SCB-CNN, which achieves 0.991; In terms of training time, the unoptimized SCB-CNN is the shortest, followed closely by the optimized SCB-CNN, in contrast, VGG-Net and GoogLeNet exhibit significantly longer training times under the same number of epochs. Based on the above information, the optimized SCB-CNN demonstrates superior performance in the classification of SCB pattern images.

**Table 16:** The classification performance of all models.

Model	epochs	Prediction Speed(samples/S)	convergence speed	*P+*	*Time/S*
**Optimized SCB-CNN**	10 epochs	69	5	0.974	122.32
	20 epochs	69	5	0.988	262.67
	50 epochs	69	5	0.989	633.62
	100 epochs	69	5	0.991	1366.27
**Unoptimized SCB-CNN**	10 epochs	37	30	0.646	124.53
	20 epochs	37	30	0.808	261.44
	50 epochs	37	30	0.968	618.69
	100 epochs	37	30	0.979	1354.48
**VGG-Net**	10 epochs	61	15	0.687	236.37
	20 epochs	61	15	0.835	481.92
	50 epochs	61	15	0.976	1204.85
	100 epochs	61	15	0.988	2554.76
**GoogLeNet**	10 epochs	70	15	0.692	322.51
	20 epochs	70	15	0.898	657.31
	50 epochs	70	15	0.983	1639.72
	100 epochs	70	15	0.993	3293.37

## 5 Conclusion and future work

The SCB is a world cultural heritage, and it is necessary to use image processing techniques for their inheritance and innovation. This paper proposes an SCB-CNN model for the recognition and classification of SCB patterns based on SDE. Through ablation experiments, various factors influencing the model’s performance are investigated. The final experimental results show that, while achieving an average P+ of 98.8%, the training time required for the optimized model has been reduced from 1354.48 seconds to 262.67 seconds. Under the condition of 20 epochs, the P+ has increased from 80.8% to 98.8%, and under the condition of 100 epochs, the P+ has increased from 97.9% to 99.1%. In terms of inference speed, the optimized SCB-CNN achieves an average inference speed of 14.5 ms (69 images per second), while the unoptimized SCB-CNN exhibits an average inference speed of 27 ms (37 images per second). VGG-16 demonstrates an average inference speed of 16.4 ms (61 images per second), and GoogleNet has an average inference speed of 14.3 ms (70 images per second). Comparative experiments with the unoptimized SCB-CNN, VGG-Net, and GoogleNet indicate that the optimized SCB-CNN significantly reduces training time while maintaining a fast inference speed, convergence speed, and a high P+ score, showing outstanding performance in the classification of pattern images. Comparative experiments with the unoptimized SCB-CNN, VGG-Net, and GoogleNet demonstrate that the optimized SCB-CNN significantly reduces training time while maintaining fast prediction speed, convergence speed, and high P+, showing excellent performance in the classification of pattern images. Future research will focus on the following aspects: continuing to collect and analyze SCB patterns, obtaining more pattern samples, expanding categories, and achieving more pattern classifications; improving and optimizing the network structure to further enhance the classification performance.
